# MALDI MSI of MeLiM melanoma: Searching for differences in protein profiles

**DOI:** 10.1371/journal.pone.0189305

**Published:** 2017-12-08

**Authors:** Roman Guran, Lucie Vanickova, Vratislav Horak, Sona Krizkova, Petr Michalek, Zbynek Heger, Ondrej Zitka, Vojtech Adam

**Affiliations:** 1 Department of Chemistry and Biochemistry, Mendel University in Brno, Brno, Czech Republic; 2 Central European Institute of Technology, Brno University of Technology, Brno, Czech Republic; 3 Institute of Animal Physiology and Genetics, Academy of Sciences of the Czech Republic, v.v.i., Libechov, Czech Republic; Universidade de Sao Paulo, BRAZIL

## Abstract

**Background:**

Treatment of advanced cutaneous melanoma remains challenging, and new data on melanoma biology are required. The most widely accepted criteria for the prognostic evaluation of melanoma are histopathological and clinical parameters, and the identification of additional tumor markers is thus of paramount importance. Matrix-assisted laser desorption/ionization mass spectrometry imaging (MALDI MSI), an important tool in cancer research, is useful for unraveling the molecular profile of melanoma.

**Methodology/Principal findings:**

In this report, we used the melanoma-bearing Libechov minipig (MeLiM), a unique animal model that allows observation of the complete spontaneous regression of invasive cutaneous melanoma, to investigate i) the differences between melanoma and healthy skin protein profiles and ii) the proteins potentially involved in spontaneous regression. The MeLiM tissues were cryosected, histologically characterized, analyzed by MALDI MSI, and immunohistologically stained. Multivariate statistical analyses of the MALDI MSI data revealed ten relevant *m/z* ions, of which the expression levels varied significantly among the studied MeLiM tissues. These ion peaks were used to create mass ion images/maps and visualize the differences between tumor and healthy skin specimens, as well as among histologically characterized tissue regions.

**Conclusions/Significance:**

Protein profiles comprising ten statistically significant mass ion peaks useful for differentiating cutaneous melanoma and healthy skin tissues were determined. Peaks at *m/z* 3044, 6011, 6140 and 10180 were overexpressed in melanoma compared with healthy skin tissue. More specifically, *m/z* 6140 was expressed at significantly (*p* < 0.05) higher levels in normally growing melanoma regions than in regions with early and late spontaneous regression. This study demonstrates the clinical utility of MALDI MSI for the analysis of tissue cryosections at a molecular level.

## Introduction

Melanoma is one of the most aggressive forms of cancer, being responsible for 1.2% of all cancer deaths in the European Union. Cancerous growth develops when unrepaired DNA damage to skin cells (most often caused by ultraviolet radiation from sunlight or tanning beds) triggers mutations that cause the skin cells to multiply rapidly and form malignant tumors [1]. Cancer cells and/or the surrounding microenvironment generate proteins and peptides of different types and in different concentrations than normal cells during cancer development [2]. Protein changes associated with the transition from melanocyte to atypia or dysplasia and ultimately to melanoma could be used to aid in diagnosis or to screen high-risk patients. Proteins associated with pathophysiology and malignant properties could be used to further classify melanoma, stratifying patients by risk of recurrence in order to better select surgical and adjuvant treatments [3]. Beyond the diagnostic and prognostic value in assessing melanoma progression, these protein signatures may provide valuable insights into the choice of optimal treatment strategies for individual patients. The most widely accepted criteria for the prognostic evaluation of melanoma are histopathological and clinical parameters, and the identification of additional tumor markers is thus of paramount importance.

Matrix-assisted laser desorption/ionization mass spectrometry imaging (MALDI MSI) is a label-free technique for spatially resolved molecular analysis of tissue sections [4, 5]. MALDI MSI has a high potential to support histopathological diagnosis [6], and increasing research activity has been aimed at establishing clinical applications of this technology [7]. Furthermore, it is a valuable method for the identification of biomarkers and can complement histology, immunohistochemistry and molecular pathology in various fields of histopathological diagnostics, especially with regard to the identification and grading of tumors [8]. Many studies have demonstrated that MALDI MSI can be used for biomarker discovery [9–11] and, more importantly, for tumor classification [12, 13] and the determination of the origin of the primary tumor [14].

Spontaneous animal tumors appear to be highly suitable models with which to study human oncology and cancer therapy [15]. The melanoma-bearing Libechov minipig (MeLiM) strain of miniature pigs with heritable cutaneous melanoma is an original animal cancer model with histopathological, biochemical and molecular biological similarities to human melanoma [16, 17]. The MeLiM strain allows observation of the complete spontaneous regression of invasive cutaneous melanoma. Thus, it is an excellent model for discovering biomarkers possibly involved in this process. A recent study applied laser ablation inductively coupled plasma mass spectrometry (LA ICP MS) to spatially map metals in histologically specified regions in MeLiM melanoma [18]. The levels of Zn and Cu in given regions varied, suggesting a connection with the expression of metal-binding proteins such as tyrosinase, Tyrp 2/Dct or metallothioneins (MTs) [18]. The abnormal distribution of proteins in tissue can be analyzed by MALDI MSI, and the patterns may help to identify cancer-specific changes (compared with controls) that may prove to be clinically useful [2].

The goal of the present study was to compare the expression levels of particular proteins/peptides in very small, histologically uniform and mutually distinct regions of melanoma samples, providing an efficient method for monitoring changes in protein levels during spontaneous regression in the MeLiM model.

## Materials and methods

### Materials

Sinapinic acid (SA), 2,5-dihydroxybenzoic acid (DHB) and all solvents (HPLC grade) used were purchased from Sigma-Aldrich (MO, USA), unless otherwise noted. Conductive indium-tin oxide (ITO) one-side coated glass slides and peptide and protein calibration standards were purchased from Bruker Daltonik GmbH (Germany).

### Sample collection

Two MeLiMs (both 10 weeks old) with multiple skin nodular melanomas (20–38 mm in size) were used in this pilot experiment. One melanoma was excised from each animal under total anesthesia [premedication with intramuscular (i.m.) administration of 0.5 mg.minipig^-1^ atropine (Hoechst-Biotika, Slovak Republic), followed by 1 mg.kg^-1^ of body weight Stresnil (Janssen Pharmaceutica N.V., Belgium) and Narcotan inhalation (Leciva, Czech Republic)]. The wound was closed by individual stitches, and Vetalgin (0.5 mg.kg^-1^ of body weight; Intervet International, Germany) was administered i.m. after the surgery was finished and for two days thereafter to control pain after tumor excision and wound suturing. The overall health status, food intake, physical activity and wound healing were monitored in both experimental animals twice a week for 2 weeks after tumor excision. The wounds were completely healed at that time, so the stitches were removed. This experimental treatment was performed in accordance with the rules of the European Convention for the Care and Use of Laboratory Animals and on the basis of the Project of Experiment that was assessed and recommended by the Expert Committee of the IAPG AS CR, v.v.i. (Libechov, Czech Republic) for ensuring the welfare of experimental animals and approved by the Expert Committee of the Academy of Sciences of the Czech Republic.

## Sample preparation

Tissue blocks were prepared according to the following protocol, which were suggested by Anyz et al. [18]. Serial cryosections (8 μm thickness for histology and immunohistochemistry and 6–10 μm thickness for MALDI MSI optimization) were prepared by a Leica CM 1850-Cryostat (Leica, Germany). Cryosections for MALDI MSI were mounted onto ITO glass slides and stored at -80°C. Prior to analysis, the slides were warmed by hand and desiccated under vacuum for 15 min. Then, the slides were washed in a Coplin jar with ethanol (twice in 70% ethanol for 2 min and once in 100% ethanol for 2 min), followed by several washings with cold ACS-grade water (10 short dips) and subsequent washing with ethanol as indicated. The matrix application samples were dried under vacuum for 15 min, and the positions of the tissue slices were marked with three guide marks using a white pencil corrector. Afterwards, the glass slides were scanned by an Epson Perfection V500 Office scanner (Epson Europe B.V., Netherlands) at a resolution of 2400 DPI.

### Histology

**Fig 1:**
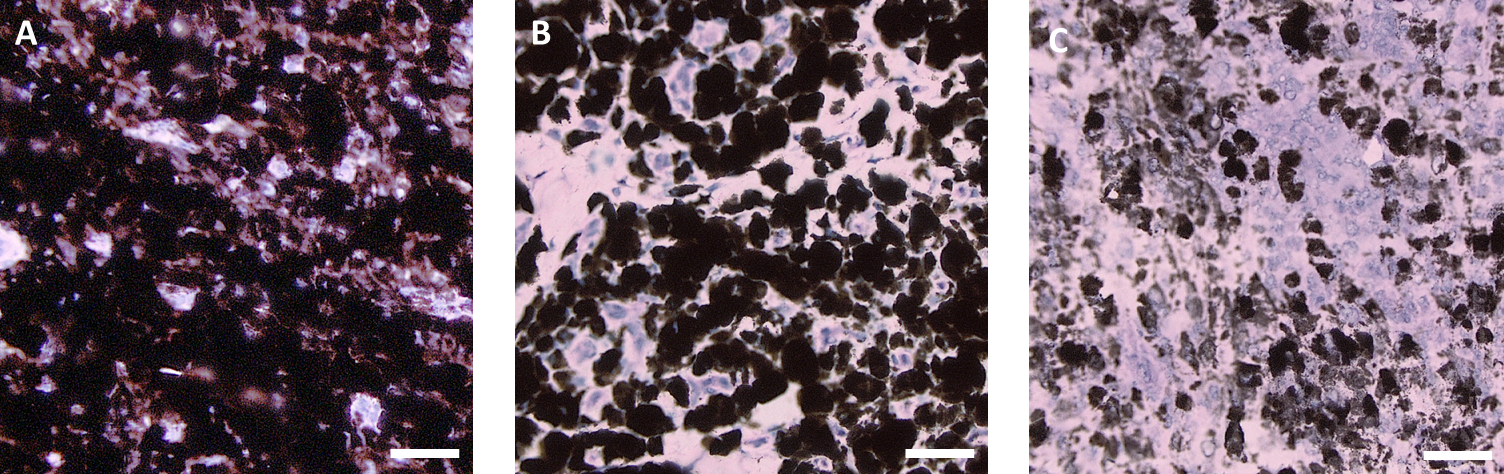
Histologically differing regions identified in hematoxylin-eosin-stained skin melanoma of a 10-week-old MeLiM. (A) Growing melanoma tissue (GMT), (B) melanoma tissue with early spontaneous regression (ESR), and (C) melanoma tissue with late spontaneous regression (LSR) (scale bar = 20 μm).

Hematoxylin-eosin staining was applied to observe tissue structure. Cryosections were fixed with ethanol (20 min), washed with distilled water (three times, 5 min each) and treated with Weigert’s hematoxylin (20 min) for nuclei staining. Then, the cryosections were washed with running tap water (20 min), followed by distilled water (three times, 5 min each). The cytoplasm was counterstained with 1% eosin alcoholic solution (1 min). After washing with distilled water (three times, 5 min each), the stained sections were embedded in glycerin jelly. Whole cryosections were scanned by a VS120 Olympus microscope with OlyVIA software (Olympus, Japan). In the scanned images, GIMP 2.8 software (http://www.gimp.org/) was used to identify three histologically different regions of melanoma tissue. Zones of growing melanoma tissue (GMT) were made up of darkly pigmented intact melanoma cells in close proximity to one another (Fig 1A). Zones of early spontaneous regression (ESR) showed lymphocyte infiltration that resulted in the destruction of some melanoma cells and the formation of large extracellular spaces. However, most melanoma cells were still well preserved (Fig 1B). In regions of late spontaneous regression (LSR), most melanoma cells were already destroyed, forming extensive cellular debris. Intact melanoma cells were only sparsely dispersed (individually or in small groups), and initial rebuilding of tumor tissue in the fibrous tissue was locally observed (Fig 1C). In healthy normal black skin, six standard histological structures were monitored—epidermis, dermis, hair follicles, sweat glands, subcutaneous adipose tissue, and subcutaneous muscle tissue. Three to six rectangular areas of each region per cryosection were chosen for comparison of the protein maps to detect the local protein contents.

### Matrix application

MALDI matrix was sprayed onto ITO glass slides using ImagePrep^TM^ standard programs (Bruker Daltonik GmbH, Germany). The following MALDI matrices were used: 10 mg.ml^-1^ SA in acetonitrile/water (60:40, v/v) with 0.2% trifluoroacetic acid (TFA) and 30 mg.ml^-1^ DHB in methanol/water (50:50, v/v) with 0.2% TFA. The MALDI matrix mixtures were thoroughly vortexed and ultrasonicated in a Bandelin 152 Sonorex Digital 10P ultrasonic bath (Bandelin electronic GmbH, Germany) for 2 min at 50% intensity at room temperature (RT). The slides were ready for analysis after drying in a vacuum desiccator for 15 min.

### MALDI MSI analysis

MSI was performed on a Bruker ultrafleXtreme MALDI tandem time-of-flight (MALDI-TOF/TOF) mass spectrometer (Bruker Daltonik GmbH, Germany). The total sample set consisted of 12 ITO glass slides containing 24 tissue sections. The scanned images of tissue slices were loaded into FlexImaging 3.0 software (Bruker Daltonik GmbH, Germany), and a MALDI adapter with two ITO glass slides was loaded into the mass spectrometer. The position of the MALDI adapter was adjusted according to the white guide marks on the ITO glass slides. The regions of acquisition were highlighted by the mouse pointer in FlexImaging, and 50 μm raster width was chosen. External calibration was performed using a peptide and protein standard mixture in an *m/z* range of 1–20 kDa. The intensity of each scan over the entire acquired mass range was mapped on the tissue section image to visualize the location of each *m/z* value detected. These images were generated and visualized using SCiLS Lab 2014b software (SCiLS–Bruker Daltonik GmbH, Germany). The laser power was set to 85% for the SA matrix and 80% for the DHB matrix. MALDI MSI of proteins was performed in linear positive mode in a *m/z* range of 1-20 kDa. A total of 500 spectra were summed for each spot using a random walk raster pattern, with no evaluation criteria. Prior to this study, the tissue thickness and laser shots per raster spot were optimized. Sections of various thickness (from 6 to 10 μm), rasters of various dimensions (30×30, 50×50, 100×100 and 200×200 μm) and different numbers of laser shots per raster spot (300, 500, 1000 and 1200 laser shots per raster spot) were tested.

### Spectral processing and statistics

The MSI data from FlexImaging were converted and uploaded into the SCiLS Lab software used for pipeline preprocessing, segmentation and statistical analysis [namely, principal component analysis (PCA), the Anderson-Darling normality test and the Kruskal-Wallis test]. The preprocessing pipeline applied baseline removal using the iterative convolution algorithm with sigma = 20 and iterations = 20. Subsequently, the total ion current (TIC) of every spectrum was normalized to one. At the same time, the mean spectrum was calculated. The segmentation pipeline applied the spectral group resulting from the preprocessing pipeline to find peaks using orthogonal matching with sigma estimated from the mean spectrum. This process found a maximum of 50 peaks per spectrum and was applied to every 15th spectrum. The consensus threshold was 1%. Then, a peak alignment was applied to the resulting histogram peak list together with the mean spectrum. Finally, the denoising and segmentation pipeline was applied to the spectral group produced by the preprocessing pipeline, and the peak list was produced by the peak alignment. The denoising level was the same as that selected in the settings of the segmentation pipeline.

The Anderson-Darling normality test revealed that the distribution of MSI data was not normal (*p* < 0.001). Therefore, the Kruskal-Wallis test was used for all hypothesis tests. The significance of differences in the peak intensities of selected masses between healthy skin and melanoma, between each region of interest (ROI) on healthy skin and between each ROI of melanoma was tested.

PCA of the MSI data of peaks selected in the segmentation pipeline was performed after the data were normalized using the preprocessing pipeline. Unit variance scaling was used.

An intensity box plot chart conveniently depicts the intensities of a given *m/z* range in the spots of a given region through their quartiles. Every box describes a single *m/z* range and a single region. Every plot consists of two parts: a box and a cloud. The box contains a rectangle divided by a horizontal line, which represents the median intensity. The median intensity is defined such that the number of spots with lower values than the median intensity is equal to the number of spots with higher intensity. The lower and upper bounds of the box represent the second and third quartile, respectively, which means that the total number of spots with intensities below these lines are one-quarter and three-quarters, respectively. The lines extending vertically from the boxes (whiskers) represent the lower and upper quantiles, respectively, which are 0.0% and 99.0%, respectively, by default. The cloud part of the plot shows how spots in a given region are spread by the intensity of a given *m/z* range. Blue dots represent the spots in which the intensities of a given *m/z* range are between the lower and upper quantiles. Red dots represent the spots with intensities outside of these intensity intervals, so-called “outliers”.

### MSI image preparation

Final preparation of the MSI images was carried out in SCiLS Lab software. According to PCA and the Kruskal-Wallis test, ion peaks with significant variability between melanoma and healthy skin tissues were used to create the MSI images. Each *m/z* value was displayed as “*m/z* ± 1.5 Da”. Final corrections and preparations of the MALDI MSI images were carried out in GIMP 2.8 software.

## Immunohistochemistry

The cryosections were incubated for 10 min at RT with phosphate-buffered saline (PBS). Endogenous peroxidase activity was blocked with 3% H_2_O_2_ in PBS for 15 min. After washing with dH_2_O, heat-induced epitope retrieval was performed using citrate buffer (10 mM, pH 6.0). After washing in PBS, the sections were incubated first with anti-MT-1 and MT-2 antibody (Dako, M0639, 1:50) for 1 hr at RT, followed by incubation with horseradish peroxidase (HRP)-conjugated rabbit anti-mouse antibody (Dako, P0260, 1:100) for 15 min at RT. The sections were developed with the 3-amino-9-ethylcarbazole (AEC) Substrate Kit, counterstained with hematoxylin and, after drying, mounted with aqueous mount.

## Results

### MALDI MSI analysis

**Fig 2:**
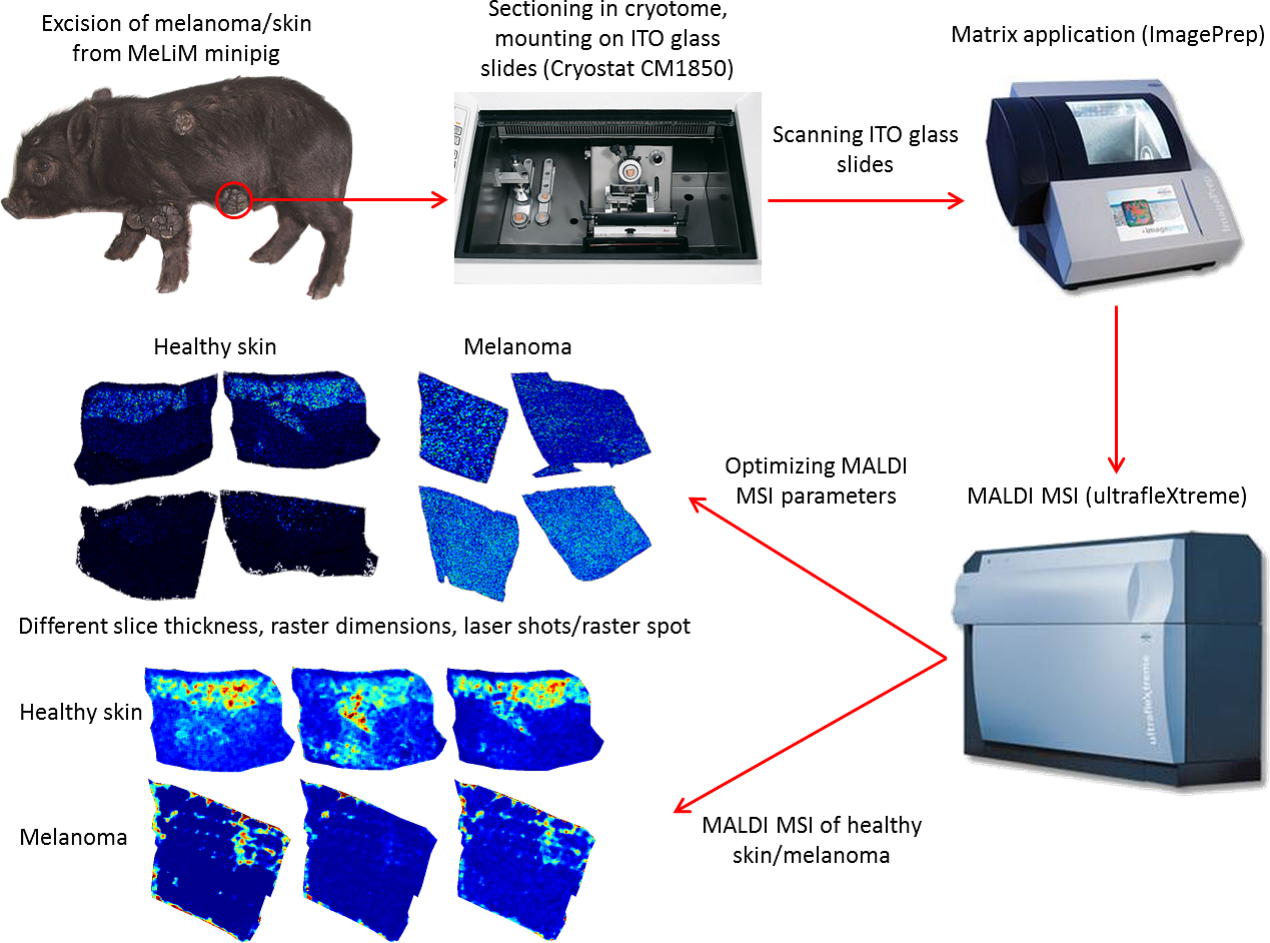
Schematic of MALDI MSI of MeLiM healthy skin/melanoma tissue.

Fresh frozen melanoma tissues from two MeLiMs were analyzed using MALDI MSI, and the resulting data were compared with data from matching healthy skin tissues stored under the same conditions. The samples were processed using a solvent washing step prior to peptide/protein MSI analysis. The MSI data were then imported into SCiLS Lab software for post-processing and generation of protein profiles and specific ion maps. The MSI data obtained after the application of an SA matrix and/or a DHB matrix was not significantly different (*p* < 0.05). Utilization of tissues with thicknesses of 8 and 10 μm resulted in satisfactory sensitivity and did not influence the variability of the spatial distribution within a tumor. The optimal number of laser shots per raster spots was found to be at least 500, and the optimal raster spot dimension was 50×50 μm. A schematic experimental workflow of the MALDI MSI experiment is provided in Fig 2.

### Statistical analysis of MALDI MSI spectra

**Fig 3:**
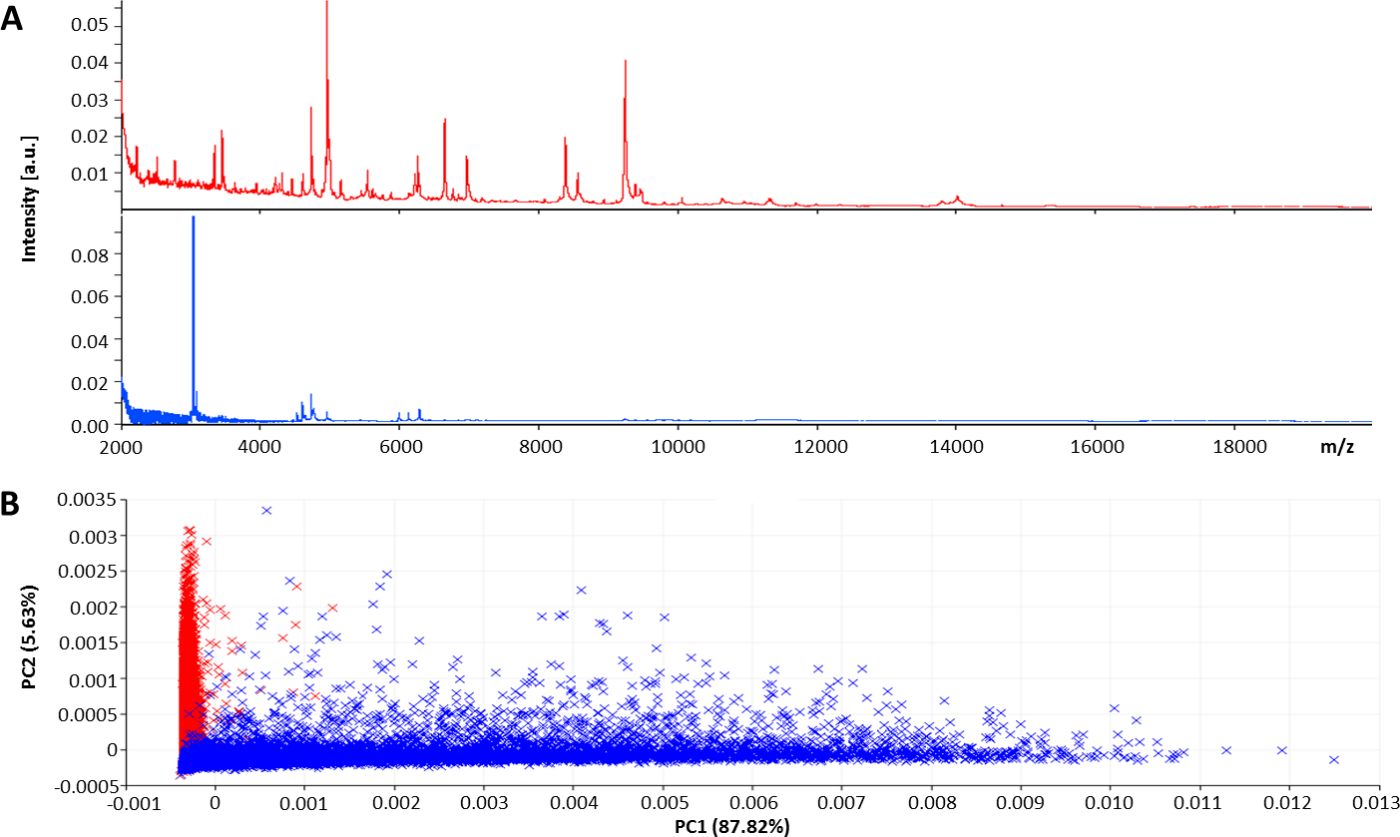
A) Average mass spectra and B) PCA scores plot of MeLiM melanoma (blue) and healthy skin (red) tissue cryosections.

The MALDI MSI spectra obtained from tissue cryosections were submitted to SCiLS Lab software to compare the average mass spectra from both tissues (Fig 3A). PCA visualized the variances between melanoma and healthy skin tissue sections, and the Kruskal-Wallis test revealed a series of significant (*p* < 0.001) ion peaks that accounted for the variation (Fig 3B, Table 1).

**Table 1:** Tentative identification of proteins with significant variation between MeLiM melanoma and healthy skin tissue.

*m/z*	Tentative identification	Rating–Kruskal-Wallis	Melanoma	Healthy skin	Reference
**3044**		<0.001	*	-	
**3458**		<0.001	-	*	
**4737**	Thymosin β-10	<0.001	-	*	[19]
**4967**		<0.001	-	*	
**6011**	Metallothionein	<0.001	*	-	
**6140**	Metallothionein	<0.001	*	-	
**6654**		<0.001	-	*	
**6985**		<0.001	-	*	
**9258**		<0.001	-	*	
**10180**	S100-A6	<0.001	*	-	UniProtKB–P06703

(*) Higher expression.

**Fig 4:**
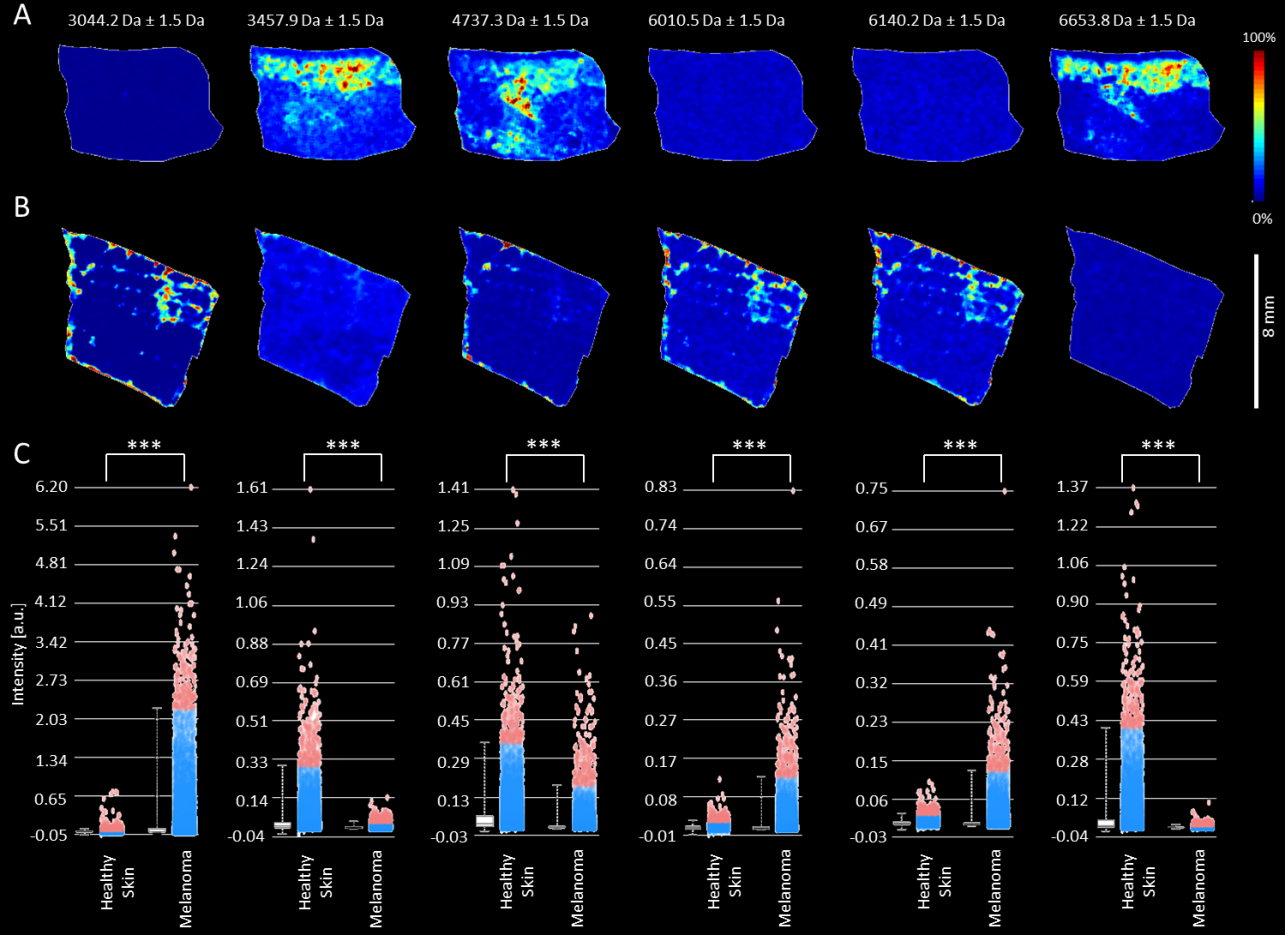
**MALDI ion images of healthy skin (A) and melanoma (B) tissue from MeLiM cryosections and intensity box plots (C) of selected *m/z* values.** The peaks at *m/z* 3044, 6011 and 6140 were overexpressed in melanoma, and those at *m/z* 3458, 4737 and 6654 were pronounced in healthy skin tissue. (***) *p* < 0.001.

The mass ion peaks at *m/z* 3044, 3458, 4737, 4967, 6011, 6140, 6654, 6985, 9258 and 10180 appeared in both types of MeLiM tissues. These ion peaks were used to generate MSI images and intensity box plots to visualize the differences between the melanoma and healthy skin tissue sections (Fig 4, S1 and S2 Figs). The intensities of several mass ion peaks differed between the two tissue types and revealed the heterogeneity of melanoma tissue. In particular, the peaks at *m/z* 3044, 6011, 6140 and 10180 were overexpressed in melanoma (S1 Fig, Table 1), and those at *m/z* 3458, 4737, 4967, 6654, 6985 and 9253 were pronounced in healthy skin tissue (S2 Fig, Table 1).

**Fig 5:**
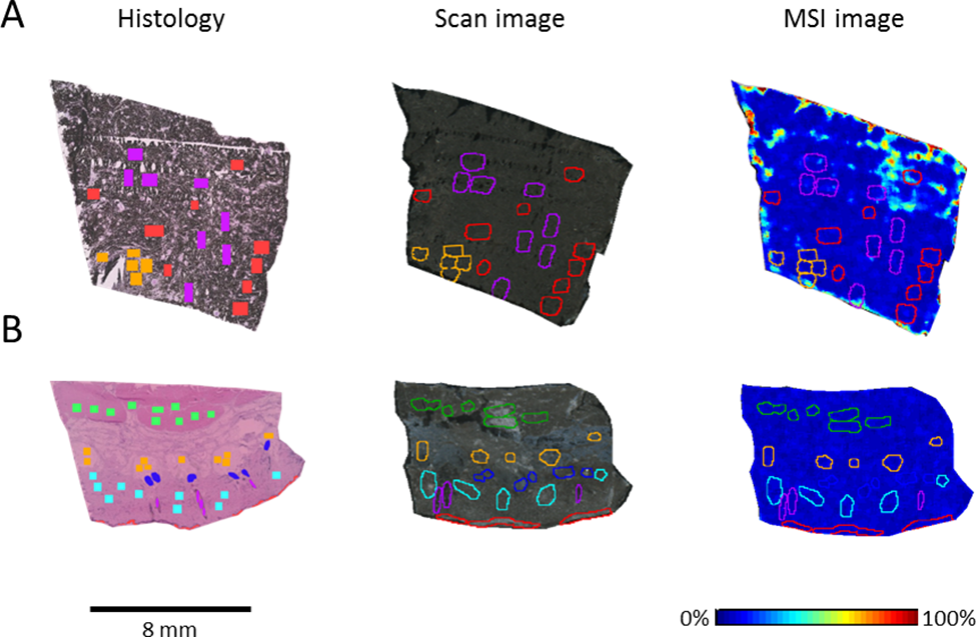
Histology, scan and MSI image of the mass ion peak at *m/z* 6011 with specified regions of differentiated tissue sections (differences between MeLiM melanoma and healthy tissue). A) Three histologically differing regions identified in hematoxylin-eosin-stained skin from porcine melanoma: red–normally growing melanoma tissue (GMT), violet–early spontaneous regression (ESR), and orange–late spontaneous regression (LSR). B) Six ROIs determined in healthy skin tissue: red–epidermis, light blue–dermis, violet–hair follicle, dark blue–sweat gland, orange–subcutaneous adipose tissue, and green–subcutaneous muscle.

Each melanoma tissue section was split into different ROIs according to the histologically uniform and mutually distinct regions of normally GMT, ESR and LSR (Fig 5A). The following ROIs were assigned in the normal skin tissue section: epidermis, dermis, hair follicle, sweat gland, subcutaneous adipose tissue and subcutaneous muscle (Fig 5B). The total spectra of these ROIs were analyzed via PCA and the Kruskal-Wallis test in SCiLS Lab software. Ion peaks with high variability were then used to create the ion images and intensity box plots (S1 and S2 Figs).

**Fig 6:**
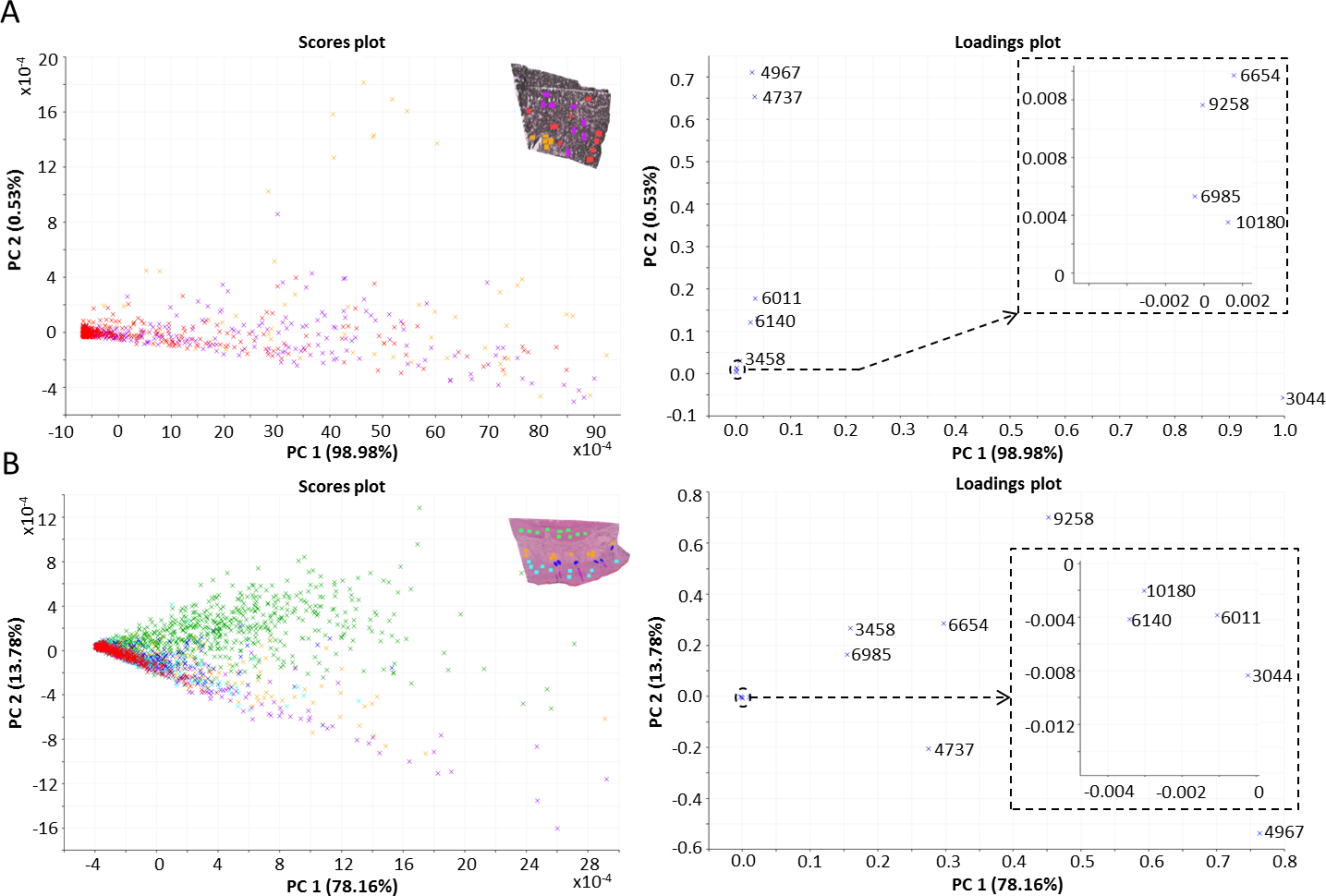
A) PCA scores and loadings plots of three histologically differentiated regions of MeLiM melanoma (inset). Red–normally growing melanoma tissue (GMT), violet–early spontaneous regression (ESR), and orange–late spontaneous regression (LSR). B) PCA scores and loadings plots of six ROIs in healthy skin tissue (inset). Red–epidermis, light blue–dermis, violet—hair follicle, dark blue–sweat gland, orange–subcutaneous adipose tissue, and green–subcutaneous muscle.

The PCA scores and loadings plots of the tumor and healthy skin ROIs are presented in Fig 6. The first two axes represented 99.51% of the total data variance. To further investigate the differences between the three histologically specified regions within the melanoma tissue, the Kruskal-Wallis test was applied (S1 Table). Ion peaks at *m/z* 3044, 3458, 4967 and 10180 varied significantly (*p* < 0.05) between the ESR and LSR regions. Comparing the GMT and ESR regions, the most variable ion peaks were located at *m/z* 3458 and 6140, while the GMT and LSR regions differed significantly (*p* < 0.01) in the expression of ion peaks at *m/z* 3044, 3458, 4737, 4967, 6140 and 10180 (Fig 6A and S1 Fig). Significant correlations were found between the ion peaks at *m/z* 6011 and 6140 (*p* < 0.05, r = 0.79) and *m/z* 6984 and 6653 (*p* < 0.05, r = 0.72) (S3 Fig). It is probable that the peaks at *m/z* 6011 and 6140 belong to same molecule; the *m/z* difference of 129 could correspond to one glutamic acid molecule. In the healthy skin tissue, PCA revealed a separation of the subcutaneous muscle region from the regions of hair follicle, subcutaneous adipose tissue and dermis (Fig 6B). The first two PC axes together accounted for 91.94% of the variance. The ion peaks at *m/z* 3458, 4737, 4967, 6140, 6654, 6985, 9258 and 10180 varied significantly (*p* < 0.001) among the six histologically specified regions within the healthy skin tissue. Notably, *m/z* 6011 did not vary significantly (*p* ≥ 0.05) in the healthy skin tissue (S2 and S3 Tables). The ion peaks at *m/z* 6011 and 6140 were tentatively identified as MTs. To verify this finding, immunohistochemical detection of MT-1 and MT-2 was performed (S4 Fig). In healthy skin, MT positivity was detected in the dermis and epidermis, which is consistent with the MSI results. However, due to strong pigmentation, no positivity or negativity for MT could be detected in the melanoma tissue.

## Discussion

The protein signatures of melanoma and healthy skin exhibited different characteristics, which is in accordance with the previously published studies. More differentially expressed proteins were observed at higher intensity in the healthy skin tissues, whereas fewer proteins with higher intensities were expressed in MeLiM melanoma specimens. In total, the intensities of ten ion peaks, *m/z* 3044, 3458, 4737, 4967, 6011, 6140, 6654, 6985, 9258 and 10180, significantly differed between melanoma and healthy skin tissue. Four ion peaks, *m/z* 3044, 6011, 6140 and 10180, were more highly expressed in melanoma tissue. The molecular weight of cysteine-rich metal-binding proteins, MTs, is approximately 6–7 kDa, which correlates with the characteristic mass peaks at *m/z* 6011 and 6140 observed here in MeLiM melanoma tissues. The calculated masses of porcine MTs according to the UniProtKB database are 5969 Da for MT-1A, 5970Da for MT-2A, 5998 Da for MT-2B, 5969 Da for MT-1E, 5955 Da for MT-1F, 5959 Da for MT-1C, 5975 Da for MT-1D and 6927 Da for MT-3 (UniProt entries P49068, P79379, P79380, P79431, P79378, P79376, P79377, and P55944, respectively). Based on a comparison of the obtained results, *m/z* 6011 and 6140 may be partially metalated MT-1 or MT-2, as metals might be partly removed from the MT molecule after interaction with the matrix solution due to the low pH [20]. Furthermore, the intensity of *m/z* 6140 varied significantly between the ESR and LSR regions and the GMT region. This peak, together with *m/z* 6011 and 3044, was overexpressed in the GMT region. Moreover, the overexpression of *m/z* 6011 and 6140 in the GMT region agrees with the higher content of zinc ions detected in the same region in our previous study [18]. These findings are in accordance with our assumption that the peaks at *m/z* 6011 and 6140 correspond to MT. MT expression by tumor cells may increase the development of melanoma, resulting in increased tumor survival and thus increased carcinogenesis. MT can reduce tyrosinase activity and melanogenesis in melanocytes, suggesting that the presence of MT prior to tumor initiation may be protective against tumor development [21]. MT overexpression has been shown to be more significantly associated with tumor progression than the established surgical prognostic test based on a sentinel lymph node biopsy [22]. A positive correlation between MT expression and tumor thickness, invasive capacity, and a higher risk of progression was previously reported [22–24]. Specifically, overexpression of MT has been found in human cutaneous malignant melanomas associated with a poor prognosis [24–26]. Moreover, overexpression of a positive MT regulator (cancer-testis antigen 16) has been shown in melanoma metastasis [27]. In the MeLiM tumor tissue, an elevated total MT content (500 μg of MTs per gram of tissue) was previously identified by an adsorptive transfer stripping technique, the Brdicka reaction [28].

In this work, MT-1 and MT-2 expression in healthy skin correlated with the localization of mass peaks *m/z* 6011 and 6140. However, in melanoma tissue, immunohistochemical detection was not possible due to the high melanin content, which interferes with staining. Several procedures for melanin bleaching of tissue sections have been published but with compromised results. Demelanization of extremely pigmented samples often leads to destruction of the tissue antigen and architecture and even to degradation of the chromogen precipitate [29]. As natural pigmentation is a frequent problem in immunohistochemistry, new non-optical detection methods are desired. Mass-spectrometry methods, either label-free methods such as MALDI MSI or employing metal labels such as laser-induced breakdown spectroscopy (LIBS) or ICP-MS, have the potential to overcome this difficulty [30–32]. Several tissue markers, including S100, MART-1, gp100/HMB45 and/or MT, are used to help distinguish melanoma from other types of cancer [26]. To date, however, there are no tissue-based biomarkers that are utilized clinically for prognostic classification [3]. In the GMT regions of the MeLiM tissues, we observed higher expression of *m/z* 6011 and 6140, which were positively correlated with each other and were assigned to MTs. Modified MT expression was previously determined in human tumors and was correlated with tumor invasiveness and a poorer prognosis, as mentioned above.

In histology-directed MALDI MSI profiling of human melanoma lymph node tissue, Hardesty and coworkers identified overexpression of several ion peaks, e.g., at *m/z* 3744, 4737, 8963 and 10625, compared with cancer-free lymph nodes [19]. These authors used a top-down approach to identify specific proteins that could be used to determine the disease stage and the inclusion of patients into either a poor or favorable group for recurrence and survival. The proteins identified in the signatures were cytochrome c, S100-A6, histone H4 and cleaved forms of thymosin β-4 and thymosin β-10 (TYB-10-2AA). In the present study, *m/z* 4737, which probably corresponds to TYB-10-2AA, was overexpressed in healthy skin tissue. Thymosin β proteins sequester actin monomers within the cell, inactivating polymerization until needed.

The S100 protein family, which is involved in Ca^2+^ homeostasis, the regulation of protein phosphorylation and cell growth and proliferation, was previously found to be expressed in early melanoma stages [33, 34]. The molecular weight of S100 proteins is approximately 10 kDa, which corresponds to the ion peak at *m/z* 10180 that was expressed in the ESR and GMT regions than in the LSR regions of the MeLiM melanoma tissue. The peak at *m/z* 10180 is probably S100-A6 protein (calcyclin), according to the UniProt database (UniProtKB—P06703). Calcyclin expression was observed in diverse cancers and was previously identified by mRNA analysis as a potential marker for aggressive melanoma [35, 36]. Moreover, calcyclin expression was higher in cells showing stronger higher Clark levels (> II), which corresponds to the vertical growth of primary melanoma cells. The overexpression of S100-A6 in the of GMT and ESR regions in MeLiM tissue confirms the findings of the abovementioned studies suggesting the use of calcyclin overexpression as an indicator of melanoma growth.

## Conclusion

This pilot study demonstrates that MALDI MSI can successfully visualize the spatial distribution of various proteins in melanoma and healthy skin tissue from MeLiM. Using suitable MSI statistical software, in our case SCiLS Lab, the significant mass peaks were detected, and the differences between melanoma and healthy skin were visualized.

In MeLiM melanoma, the mass peaks at *m/z* 3044, 6011, 6140 and 10180 were significantly (*p* < 0.001) overexpressed, whereas in MeLiM healthy skin, the mass peaks at *m/z* 3458, 4737, 4967, 6654, 6985 and 9258 were significantly (*p* < 0.001) overexpressed. The occurrence of the peaks at *m/z* 6011 and 6140 in the GMT regions was significantly (*p* < 0.05, r = 0.79) correlated; these peaks were tentatively identified as the metal-binding protein MT. The overexpression of *m/z* 6011 and 6140 was observed in the normally GMT region and correlates with the higher content of zinc ions found in the identical MeLiM tissue region [18, 28]. Moreover, the significantly (*p* < 0.05) higher expression of the peak at *m/z* 6140 in the GMT region than in the ESR and LSR regions suggests that this protein is associated with melanoma growth. The peak at *m/z* 10180 was tentatively identified as the protein S100-A6 according to the UniProtKB (P06703) database. The peak at *m/z* 4737 was tentatively identified as thymosin β-10 lacking two amino acids according to the study of Hardesty and coworkers [19]. In analyzing the MeLiM tissue by MALDI MSI, each of the tentatively identified proteins (specifically, MT, S100-A6 and thymosin β-10) could be monitored individually within a single experiment, enabling multiplex signature generation for the improvement of melanoma diagnostics.

The exact identification of the detected peaks by *on-tissue* trypsinization followed by MALDI-TOF/TOF analysis was not feasible in the present study because of the limited amount of the tissue samples. In the near future, we will focus on the exact identification of detected peaks either by MALDI-TOF/TOF or by the extraction of proteins from homogenized melanoma samples and their separation by 2D PAGE and in-gel trypsinization followed by MALDI-TOF/TOF or high-performance liquid chromatography coupled to electrospray ionization and quadrupole time-of-flight mass spectrometry (HPLC-ESI-qTOF MS) analysis.

In conclusion, our results contribute to the basic knowledge of the protein distribution in histologically specified regions in MeLiM tissues and will be used in further experiments aimed at elucidating the processes involved in spontaneous melanoma regression.

## Supporting information

S1 Fig Histology and MSI ion images of histologically specified regions of melanoma tissue from MeLiM cryosections and intensity box plots of selected *m/z* values. Red–normally growing melanoma tissue (GMT), violet–early spontaneous regression (ESR), and orange–late spontaneous regression (LSR).
(DOCX)

S2 Fig Histology and MSI ion images of histologically specified regions of healthy skin tissue from MeLiM cryosections and box plots of selected *m/z* values. Red–epidermis, light blue–dermis, violet–hair follicle, dark blue–sweat gland, orange–subcutaneous adipose tissue, and green–subcutaneous muscle.
(DOCX)

S3 Fig Correlation matrix of the ion peaks that account for the variation between melanoma ROIs. (DOCX)

S4 Fig Immunohistochemical detection of MT-1 and MT-2. Area of healthy skin corresponding to a MALDI MSI scan (A) and area of melanoma corresponding to a MALDI MSI scan (B). Detailed microphotographs of the immunohistochemical detection of MTs in healthy skin and the high melanin content in melanoma tissue (C). Arrows indicates immunopositivity; asterisk indicates melanin.
(DOCX)

S1 Table Ion peaks of interest that account for the variation among the three histologically differing regions identified in hematoxylin-eosin-stained skin of porcine melanoma. Normally growing melanoma tissue (GMT), early spontaneous regression (ESR), and late spontaneous regression (LSR). Kruskal-Wallis test. Rating: (*) *p* < 0.05, (**) *p* < 0.01, and (***) *p* < 0.001.
(DOCX)

S2 Table Ion peaks of interest that account for the variation among the six ROIs in healthy skin porcine tissue. E –epidermis, D –dermis, HF –hair follicle, SG – sweat gland, SAT – subcutaneous adipose tissue, and SM – subcutaneous muscle. *p* << 0.001 is marked as 0. Kruskal-Wallis test. Rating: (*) *p* < 0.05, (**) *p* < 0.01, and (***) *p* < 0.001.
(DOCX)

S3 Table Ion peaks of interest that account for the variation among the six ROIs in healthy skin porcine tissue. E –epidermis, D –dermis, HF –hair follicle, SG – sweat gland, SAT – subcutaneous adipose tissue, and SM – subcutaneous muscle. *p* << 0.001 is marked as 0. Kruskal-Wallis test. Rating: (*) *p* < 0.05, (**) *p* < 0.01, and (***) *p* < 0.001.
(DOCX)
