# Supplementary material for: MALDI MSI of MeLiM melanoma: Searching for differences in protein profiles
Source: PLoS One. 2017 Dec 8;12(12):e0189305. doi: 10.1371/journal.pone.0189305 (PMC5722329; doi:10.1371/journal.pone.0189305)
Supplement: S1 Fig — Red–normally growing melanoma tissue (GMT), violet–early spontaneous regression (ESR), and orange–late spontaneous regression (LSR). (DOCX) [file pone.0189305.s001.docx]

**S1 Fig. Histology and MSI ion images of histologically specified regions of melanoma tissue from MeLiM cryosections and intensity box plots of selected *m/z* values.** Red – normally growing melanoma tissue (GMT), violet – early spontaneous regression (ESR), and orange – late spontaneous regression (LSR).

**
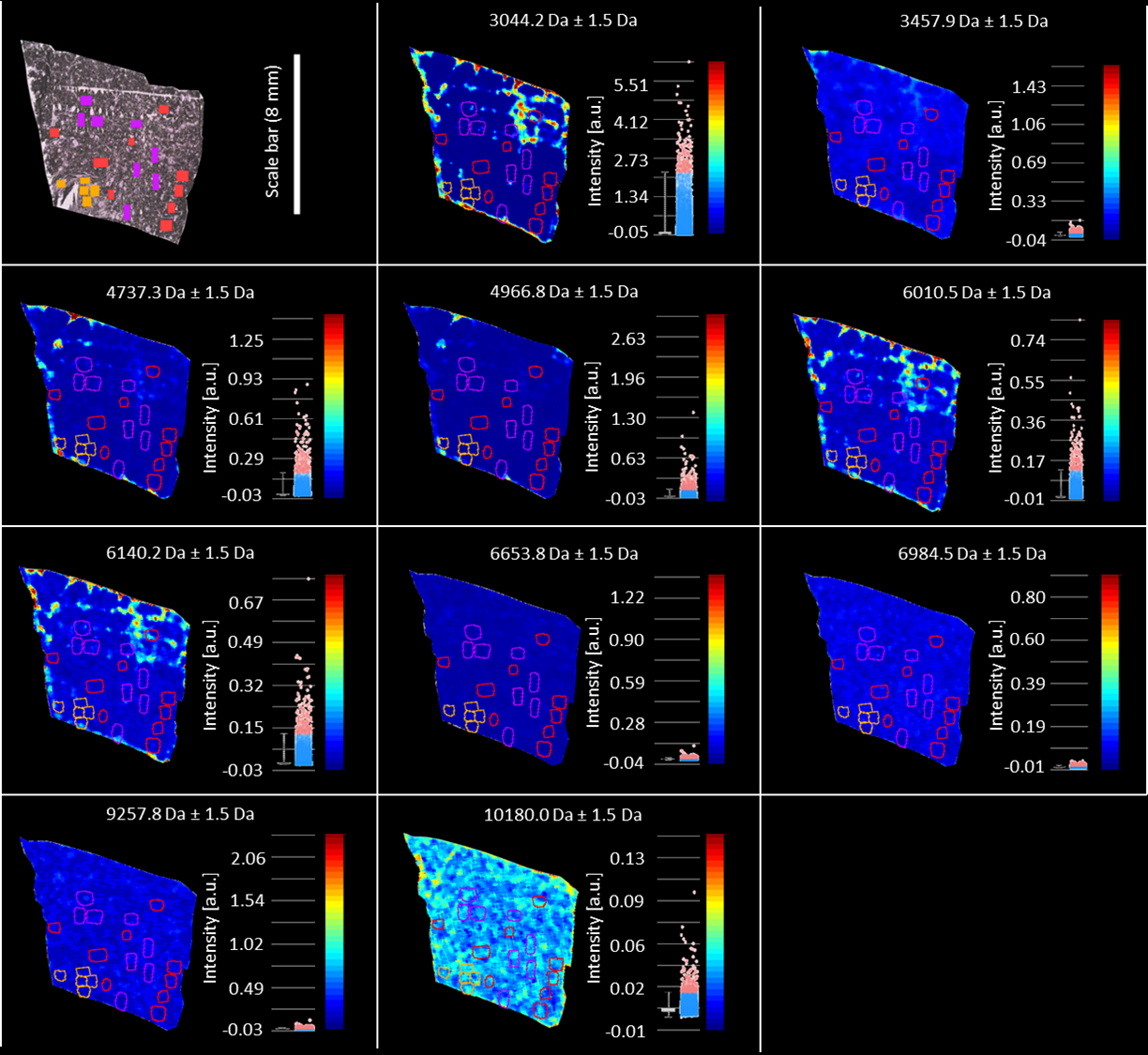
**
