# Supplementary material for: MALDI MSI of MeLiM melanoma: Searching for differences in protein profiles
Source: PLoS One. 2017 Dec 8;12(12):e0189305. doi: 10.1371/journal.pone.0189305 (PMC5722329; doi:10.1371/journal.pone.0189305)

**S2 Fig. Histology and MSI ion images of histologically specified regions of healthy skin tissue from MeLiM cryosections and box plots of selected *m/z* values.** Red – epidermis, light blue – dermis, violet – hair follicle, dark blue – sweat gland, orange – subcutaneous adipose tissue, and green – subcutaneous muscle.


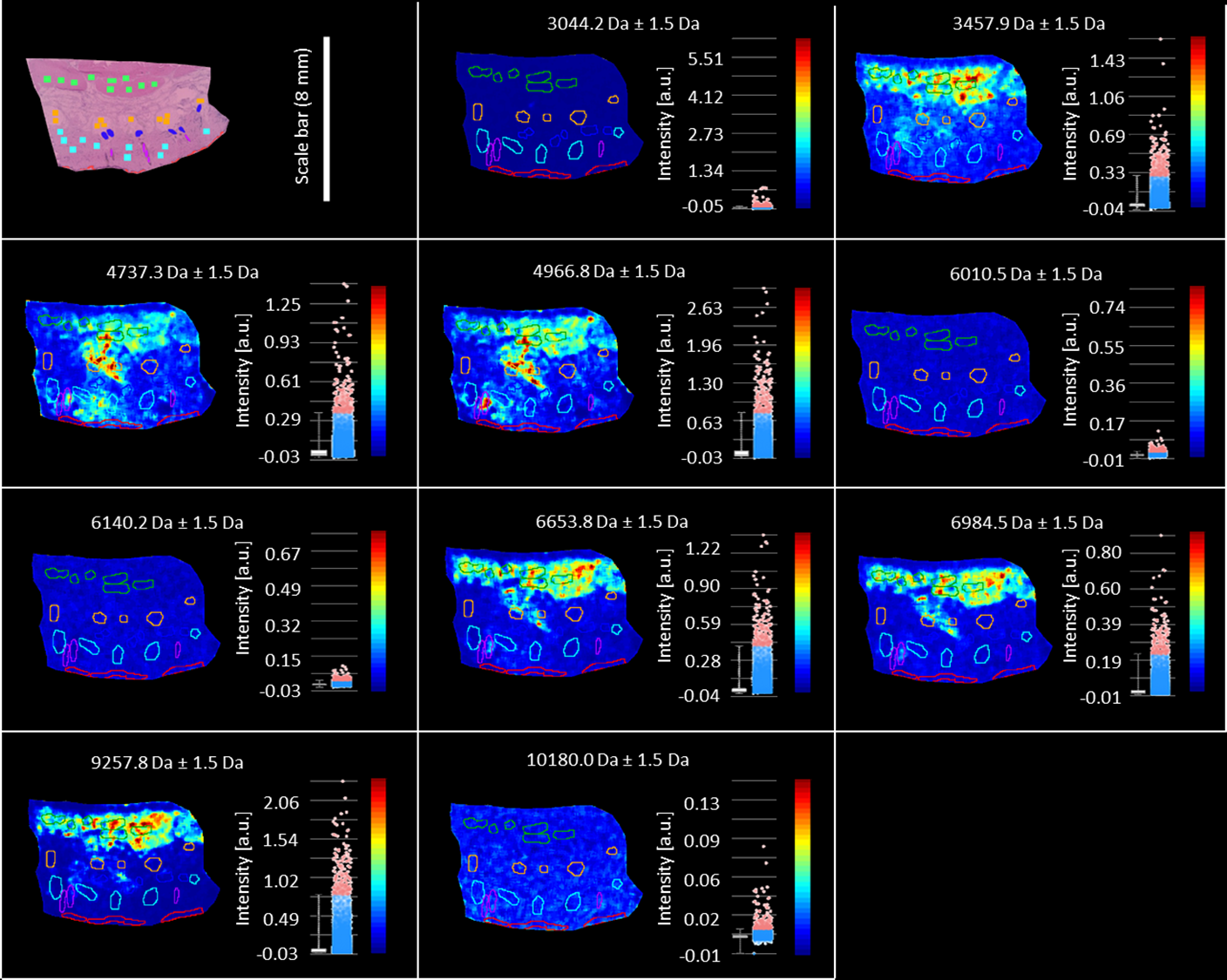

Supplement: S2 Fig — Red–epidermis, light blue–dermis, violet–hair follicle, dark blue–sweat gland, orange–subcutaneous adipose tissue, and green–subcutaneous muscle. (DOCX) [file pone.0189305.s002.docx]
