# Supplementary figures and images for: MALDI MSI of MeLiM melanoma: Searching for differences in protein profiles
Source: PLoS One. 2017 Dec 8;12(12):e0189305. doi: 10.1371/journal.pone.0189305 (PMC5722329; doi:10.1371/journal.pone.0189305)

**S3 Fig. Correlation matrix of the ion peaks that account for the variation between melanoma ROIs.**

**
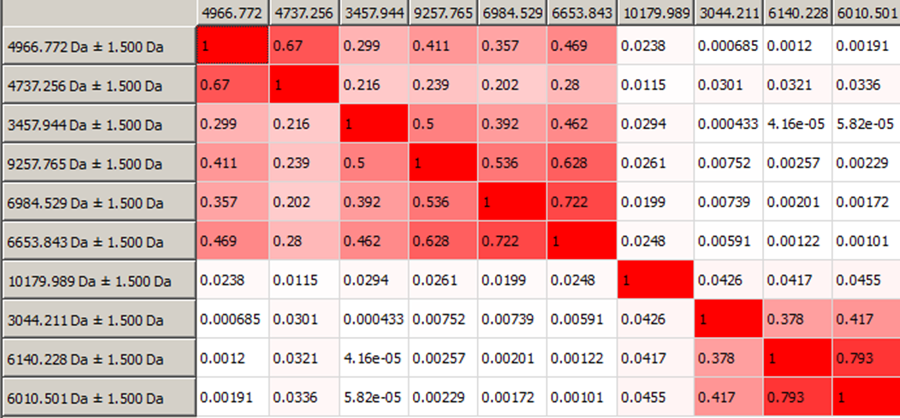
**

Supplement: S3 Fig — (DOCX) [file pone.0189305.s003.docx]
