# Supplementary material for: MALDI MSI of MeLiM melanoma: Searching for differences in protein profiles
Source: PLoS One. 2017 Dec 8;12(12):e0189305. doi: 10.1371/journal.pone.0189305 (PMC5722329; doi:10.1371/journal.pone.0189305)

**S4 Fig. Immunohistochemical detection of MT-1 and MT-2.** Area of healthy skin corresponding to a MALDI MSI scan (A) and area of melanoma corresponding to a MALDI MSI scan (B). Detailed microphotographs of the immunohistochemical detection of MTs in healthy skin and the high melanin content in melanoma tissue (C). Arrows indicates immunopositivity; asterisk indicates melanin.


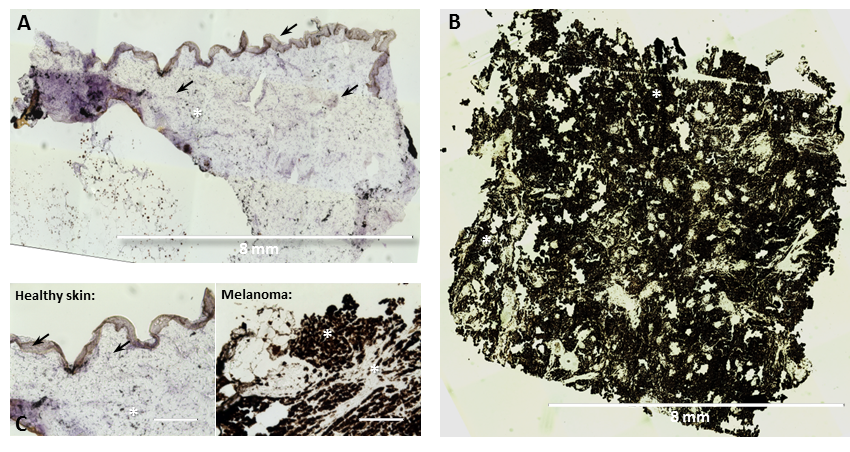

Supplement: S4 Fig — Area of healthy skin corresponding to a MALDI MSI scan (A) and area of melanoma corresponding to a MALDI MSI scan (B). Detailed microphotographs of the immunohistochemical detection of MTs in healthy skin and the high melanin content in melanoma tissue (C). Arrows indicates immunopositivity; asterisk indicates melanin. (DOCX) [file pone.0189305.s004.docx]
