# Supplementary material for: MALDI MSI of MeLiM melanoma: Searching for differences in protein profiles
Source: PLoS One. 2017 Dec 8;12(12):e0189305. doi: 10.1371/journal.pone.0189305 (PMC5722329; doi:10.1371/journal.pone.0189305)
Supplement: S1 Table — Normally growing melanoma tissue (GMT), early spontaneous regression (ESR), and late spontaneous regression (LSR). Kruskal-Wallis test. Rating: (*) p < 0.05, (**) p < 0.01, and (***) p < 0.001. (DOCX) [file pone.0189305.s005.docx]

**S1 Table. Ion peaks of interest that account for the variation between three histologically differing zones identified in haematoxylin-eosin stained skin porcine melanoma.**

| ***m/z* [Da]** | **LSR vs ESR** | | **ESR vs GMT** | | **LSR vs GMT** | | **ESR vs LSR vs GMT** | |
| --- | --- | --- | --- | --- | --- | --- | --- | --- |
|  | ***p*** | **Rating** | ***p*** | **Rating** | ***p*** | **Rating** | ***p*** | **Rating** |
| **3044** | 5,22E-06 | *** | 0,730829 | ≥0.05 | 8,86E-06 | *** | 3,53E-06 | *** |
| **3458** | 1,45E-06 | *** | 0,000519 | *** | 0,035937 | * | 4,90E-07 | *** |
| **4737** | 0,058033 | ≥0.05 | 0,14351 | ≥0.05 | 0,000932 | *** | 0,002485 | ** |
| **4967** | 0,002145 | ** | 0,733736 | ≥0.05 | 0,005018 | ** | 0,003659 | ** |
| **6011** | 0,796221 | ≥0.05 | 0,286959 | ≥0.05 | 0,395439 | ≥0.05 | 0,315412 | ≥0.05 |
| **6140** | 0,279508 | ≥0.05 | 0,014507 | <0.05 | 0,000932 | *** | 0,000961 | *** |
| **6654** | 0,76052 | ≥0.05 | 0,432967 | ≥0.05 | 0,167988 | ≥0.05 | 0,315412 | ≥0.05 |
| **6985** | 0,796221 | ≥0.05 | 0,375719 | ≥0.05 | 0,53984 | ≥0.05 | 0,427951 | ≥0.05 |
| **9258** | 0,796221 | ≥0.05 | 0,657255 | ≥0.05 | 0,837091 | ≥0.05 | 0,775275 | ≥0.05 |
| **10180** | 0,021762 | * | 0,730829 | ≥0.05 | 0,007126 | ** | 0,017228 | * |

GMT (normally growing melanoma tissue), ESR (early spontaneous regression), LSR (late spontaneous regression). Kruskal‑Wallis test was used. Used rating: (*) *p*-value < 0.05, (**) *p*‑value < 0.01, (***)*p*-value < 0.001
