# Supplementary material for: MALDI MSI of MeLiM melanoma: Searching for differences in protein profiles
Source: PLoS One. 2017 Dec 8;12(12):e0189305. doi: 10.1371/journal.pone.0189305 (PMC5722329; doi:10.1371/journal.pone.0189305)
Supplement: S3 Table — E –epidermis, D –dermis, HF –hair follicle, SG – sweat gland, SAT – subcutaneous adipose tissue, and SM – subcutaneous muscle. p << 0.001 is marked as 0. Kruskal-Wallis test. Rating: (*) p < 0.05, (**) p < 0.01, and (***) p < 0.001. (DOCX) [file pone.0189305.s007.docx]

**S3 Table.** **Ion peaks of interest that account for the variation between six regions of interest (ROIs) in healthy skin porcine tissue.**

| ***m/z* [Da]** | **E vs SM** | | **E vs SG** | | **HF vs SAT** | | **HF vs SM** | | **HF vs SG** | | **SAT vs SM** | | **SAT vs SG** | | **SM vs SG** | |
| --- | --- | --- | --- | --- | --- | --- | --- | --- | --- | --- | --- | --- | --- | --- | --- | --- |
|  | ***p*** | **Rating** | ***p*** | **Rating** | ***p*** | **Rating** | ***p*** | **Rating** | ***p*** | **Rating** | ***p*** | **Rating** | ***p*** | **Rating** | ***p*** | **Rating** |
| **3044** | 0.054 | >=0.05 | 0.009 | ** | 0.33 | >=0.05 | 0.42 | >=0.05 | 0.91 | >=0.05 | 0.86 | >=0.05 | 0.26 | >=0.05 | 0.20 | >=0.05 |
| **3458** | 0 | *** | 0 | *** | 0.003 | ** | 0 | *** | 0 | *** | 0 | *** | 0.16 | >=0.05 | 0 | *** |
| **4737** | 0 | *** | 0.002 | ** | 0.32 | >=0.05 | 0.002 | ** | 0 | *** | 0 | *** | 0.07 | >=0.05 | 0 | *** |
| **4968** | 0 | *** | 0 | *** | 0 | *** | 0.36 | >=0.05 | 0 | *** | 0 | *** | 0.36 | >=0.05 | 0 | *** |
| **6011** | 0.93 | >=0.05 | 0.44 | >=0.05 | 0.92 | >=0.05 | 0.84 | >=0.05 | 0.441 | >=0.05 | 0.91 | >=0.05 | 0.44 | >=0.05 | 0.41 | >=0.05 |
| **6140** | 0 | *** | 0.35 | >=0.05 | 0.13 | >=0.05 | 0.94 | >=0.05 | 0.21 | >=0.05 | 0.02 | * | 0.83 | >=0.05 | 0.06 | >=0.05 |
| **6654** | 0 | *** | 0 | *** | 0.13 | >=0.05 | 0 | *** | 0 | *** | 0 | *** | 0.03 | * | 0 | *** |
| **6985** | 0 | *** | 0 | *** | 0.13 | >=0.05 | 0 | *** | 0 | *** | 0 | *** | 0.06 | >=0.05 | 0 | *** |
| **9258** | 0 | *** | 0 | *** | 0.93 | >=0.05 | 0 | *** | 0.57 | >=0.05 | 0 | *** | 0.44 | >=0.05 | 0 | *** |
| **10180** | 0 | *** | 0.89 | >=0.05 | 0.007 | ** | 0 | *** | 0.44 | >=0.05 | 0.09 | >=0.05 | 0.07 | >=0.05 | 0 | *** |

E – epidermis, D – dermis, HF – hair follicle, SG – sweat gland, SAT – subcutaneous adipose tissue, SM – subcutaneous muscle. *P*-values <<0.001 are marked as 0. Kruskal-Wallis test was used. Used rating: (*) *p*-value < 0.05, (**) *p*-value < 0.01, (***) *p*-value < 0.001.
